# Experimental Usutu Virus Infection in Domestic Canaries *Serinus canaria*

**DOI:** 10.3390/v12020164

**Published:** 2020-01-31

**Authors:** Emna Benzarti, José Rivas, Michaël Sarlet, Mathieu Franssen, Daniel Desmecht, Jonas Schmidt-Chanasit, Giovanni Savini, Alessio Lorusso, Anne-Sophie Van Laere, Mutien-Marie Garigliany

**Affiliations:** 1 Fundamental and Applied Research for Animals & Health (FARAH), Faculty of Veterinary Medicine, University of Liège, Sart Tilman B43, B-4000 Liège, Belgium; benzarti_e@yahoo.com (E.B.); jfrivast@gmail.com (J.R.); Michael.Sarlet@uliege.be (M.S.); mfranssen@uliege.be (M.F.); Daniel.desmecht@ulg.ac.be (D.D.); as.vanlaere@uliege.be (A.-S.V.L.); 2 Bernhard Nocht Institute for Tropical Medicine, WHO Collaborating Centre for Arbovirus and Haemorrhagic Fever Reference and Research, 20359 Hamburg Germany; jonassi@gmx.de; 3 Faculty of Mathematics, Informatics and Natural Sciences, University of Hamburg, 20354 Hamburg, Germany; 4 OIE Reference Centre for West Nile Disease, Istituto Zooprofilattico Sperimentale “G. Caporale”, 64100 Teramo, Italy; g.savini@izs.it (G.S.); a.lorusso@izs.it (A.L.)

**Keywords:** domestic canaries, Usutu virus, experimental infection

## Abstract

Usutu virus (USUV) is a neurotropic flavivirus closely related to West Nile virus (WNV). Its enzootic cycle mainly involves mosquitoes and birds. Human infection can occur with occasional, but sometimes severe, neurological complications. Since its emergence and spread in Europe over the last two decades, USUV has been linked to significant avian outbreaks, especially among *Passeriformes*, including European blackbirds (*Turdus merula*). Strikingly, no in vivo avian model exists so far to study this arbovirus. The domestic canary (*Serinus canaria*) is a passerine, which is considered as a highly susceptible model of infection by WNV. Here, we experimentally challenged domestic canaries with two different doses of USUV. All inoculated birds presented detectable amounts of viral RNA in the blood and RNA shedding via feathers and droppings during the early stages of the infection, as determined by RT-qPCR. Mortality occurred in both infected groups (1/5 and 2/5, respectively) and was not necessarily correlated to a pure neurological disease. Subsequent analyses of samples from dead birds showed histopathological changes and virus tropism mimicking those reported in naturally infected birds. A robust seroconversion followed the infection in almost all the surviving canaries. Altogether, these results demonstrate that domestic canaries constitute an interesting experimental model for the study of USUV pathogenesis and transmission.

## 1. Introduction

Usutu virus (USUV) is a mosquito-borne virus classified together with the West Nile virus (WNV) in the Japanese encephalitis virus (JEV) serogroup, of the family *Flaviviridae*, genus *Flavivirus* [1]. It has become an endemic pathogen in many European countries and has been co-circulating with WNV, in a similar mosquito–bird life cycle, with humans and other mammals being occasional hosts [2,3,4,5]. USUV infection in humans is often asymptomatic. Nonetheless, an increasing number of cases with neurological complications, such as encephalitis or meningoencephalitis, have recently been reported [3,6,7,8,9,10,11]. In terms of animal health, USUV has been responsible for several epornitics in Europe since 1996 [12]. At least 99 European bird species, belonging to 36 different families [13,14,15,16], are currently known to be susceptible to USUV infection. However, only in a few of these avian species a fatal disease linked to USUV has been described [17], including the European blackbird (*Turdus merula*) [15,18,19,20,21], house sparrow (*Passer domesticus*) [22,23], grey owl (*Strix nebulosa*) [24], and common scoter (*Melanitta nigra*) [14]. The reasons for this selective pathogenicity are, still, unknown and avian models are critically needed to study the pathogenesis, transmission routes, and virulence of USUV.

Experimental infections of avian species with USUV are scarce and restricted so far to the domestic goose (*Anser anser f. domestica*) [25] and domestic chicken (*Gallus gallus domesticus*) [26], which were reported to be resistant to USUV infection. No experimental infection has been conducted, so far, on a passerine, although *Passeriformes* are suspected to be relevant hosts for the amplification of USUV [27], as in the case of WNV [2]. The domestic canary (*Serinus canaria*) belongs to the same order (*Passeriformes*) as the European blackbird, which is highly susceptible to USUV infection. This species is accustomed to captivity and is more convenient to use in the laboratory than wild-caught European blackbirds [28]. Further, canaries are considered as an excellent model of infection by WNV [28]. Epidemiological surveys carried out in Germany during the period between 2011 and 2013 and during 2017–2018 detected USUV genomic RNA in these birds, indicating that they can be naturally infected with the virus [19,29,30]. However, whether USUV can be pathogenic for this species is still unknown. To address this question, we experimentally challenged domestic canaries with two different doses of USUV. We investigated clinical signs, RNAemia, RNA shedding, and seroconversion in the surviving birds. In parallel, histopathological changes and virus distribution were examined in the lethally infected birds.

## 2. Materials and Methods

### 2.1. Virus and Birds

Usutu virus strain UR-10-Tm belonging to the European lineage 2 (*GenBank*: KX555624) was used in this experiment. It was isolated from a pool of organs including the brain, spleen, kidney, and heart of a blackbird found dead during an episode of anomalous mortality, which occurred in 2010 in the province of Pesaro Urbino (Italy).

The virus was amplified in African Green Monkey Vero cells (ATCC® CRL-1586; passage number 5) and titrated by the 50% tissue culture infective dose (TCID_50_) technique. 

Fifteen ten-month-old male canaries were obtained from Smet’s breeding facility (Vivegnis, Liege, Belgium; certification number: HK51603061). The birds were transported to the biosafety level 2 (BSL-2) experimental animal facility of the Department of Pathology, Faculty of Veterinary Medicine, Liège, Belgium, where they were marked by a unique colored and numerated leg band and housed in randomly-composed groups of five per cage with water and grains supplied ad libitum. One week later, all birds were blood-sampled and tested for the presence of USUV and WNV antibodies prior to the experimental infection (see section: Detection of antibodies to USUV). The animal care and procedures performed in this experiment were approved and supervised by the Committee for Ethics in Animal Experimentation of the University of Liege, Belgium (Identification code: 18-2024, date of approval: 16/08/2018).

### 2.2. USUV Challenge

Birds were assigned to three groups: control (*n* = 5), group A (high dose, *n* = 5), and group B (low dose, *n* = 5), then anesthetized via isoflurane inhalation. After weighting, groups A and B were inoculated using the intraperitoneal route with either a high dose (10^6^TCID_50_/individual) or a low dose (10^3^TCID_50_/individual) of USUV, respectively, dispersed in 100 µL of cell culture medium (Dulbecco’s Minimum Essential Medium (DMEM) supplemented with 1% penicillin/streptomycin). The control group was injected with an equivalent volume of the virus-free medium. After infection, each group was maintained in a separate wire cage with a removable floor that was cleaned daily.

### 2.3. Sample Collection

Following the challenge, birds were monitored twice daily for 15 days post-infection (dpi). A 100 µL blood sample was collected from the jugular vein of each bird at 1, 3, 9 and 15 dpi to assess the course of RNAemia and antibody response. The blood was then added to phosphate-buffered saline (PBS) in a ratio of 1:5 and allowed to clot at 4 °C. All the birds were weighed and immature feathers were collected according to the same sampling schedule to reduce stress and repetitive anesthesia. Droppings were daily collected from the cages during the first week of infection and stored at −80 °C until use. Birds that succumbed to the infection were necropsied and 50 ± 1 mg of the brain, eye tissues, lung, liver, kidney, and intestines were harvested for PCR analysis. Other portions of these organs, as well as the heart and spleen, were fixed in 4% formalin for histological and immunohistological examinations. Approximately 10 ± 1 mg of immature feathers were, also, collected from each of these birds.

### 2.4. Histopathology and Immunohistochemistry

Tissue samples preserved in formalin were embedded in paraffin wax, sectioned and then stained with hematoxylin and eosin. Slides were also processed for immunohistochemistry (IHC) as described in [23] using a mix of monoclonal anti-E protein 4E9 and 4G2 antibodies at a 1/200 dilution.

### 2.5. USUV Genome Detection

RNA was extracted from 125 µl of diluted serum and the viral genome load was measured by RT-qPCR, as described in [23]. Tissues, feathers and droppings samples were examined using the same protocol as [23]. Viral RNA copies (VRC) were calculated by absolute quantification using a standard curve, which was constructed as described in [31] using the following primers (T7 promoter-USUVF-TAATACGACTCACTATAGGAAGACATCGTTCTCGACTTTG and USUVR-CAGCACCAGTCTGTGACCAG). 

### 2.6. Detection of Antibodies to USUV

Serum samples were screened for antibodies using a competitive ELISA kit (ID Screen® West Nile Competition Multi-species, Grabels, France) following the manufacturer’s instructions. This kit is able to detect immunoglobulins M and G directed against the envelope protein of WNV, which contains an epitope common to viruses from the JEV serocomplex, including USUV [32,33]. Blood samples collected at day 15 pi were further tested for USUV-neutralizing antibodies, which primarily target the USUV envelope glycoprotein [34], using a virus neutralization test in microtiter plates (SN) as described in [35]. Neutralization titers were assigned based on the highest dilution of each serum where the complete absence of cytopathic effects in the cell monolayer was observed.

### 2.7. Statistical Analyses

Survival curves were plotted and compared using the Log-Rank and the Gehan-Breslow Wilcoxon tests (GraphPad Software, La Jolla, CA, USA).

Levels of RNAemia and virus shedding via droppings and feathers were checked for normality using Shapiro–Wilk and Kolmogorov–Smirnov statistics. The logarithmic transformation was performed to normalize the distribution of the data revealed as non-parametric. Data were then analyzed using ANOVA implemented in Rstudio. *p*-values < 0.05 were considered statistically significant.

## 3. Results

### 3.1. Survival and Body Weight Changes

**Figure 1:**
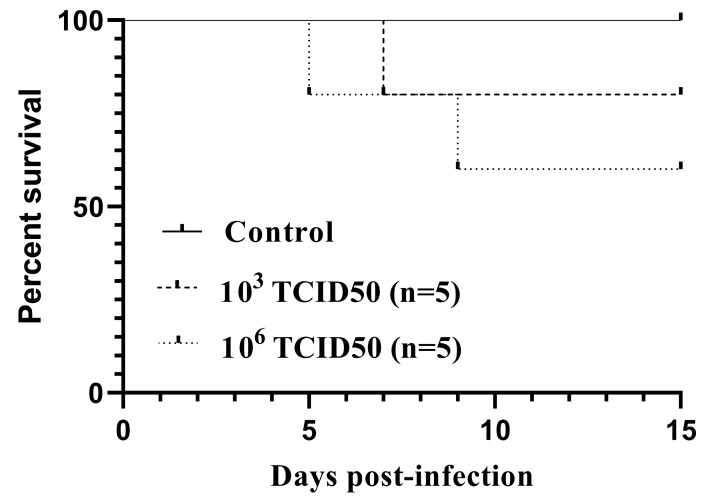
Kaplan–Meier survival curves for canaries intraperitoneally inoculated with 10^3^TCID_50_ (*n* = 5) or 10^6^TCID_50_ (*n* = 5) of the Usutu virus.

All the infected birds but one showed inactivity and fluffed feathers between days 5 and 9 pi. Two out of five birds from group A and one from group B succumbed without showing specific signs prior to death. The survival curves (Figure 1) did not differ statistically between the infected groups, as assessed by both the log-rank (Mantel-Cox) χ^2^ = 2.322, *P* = 0.3131, and the Gehan-Breslow Wilcoxon tests χ^2^ = 2.305, *P* = 0.3158. For surviving canaries, no loss in body weight was observed (data not shown). However, a loss in the initial body mass ranging from 15.9% to 19.6% was recorded in the dead birds. No weight loss or fatality was detected in the control group.

### 3.2. Necropsy and Histopathology Findings

**Figure 2:**
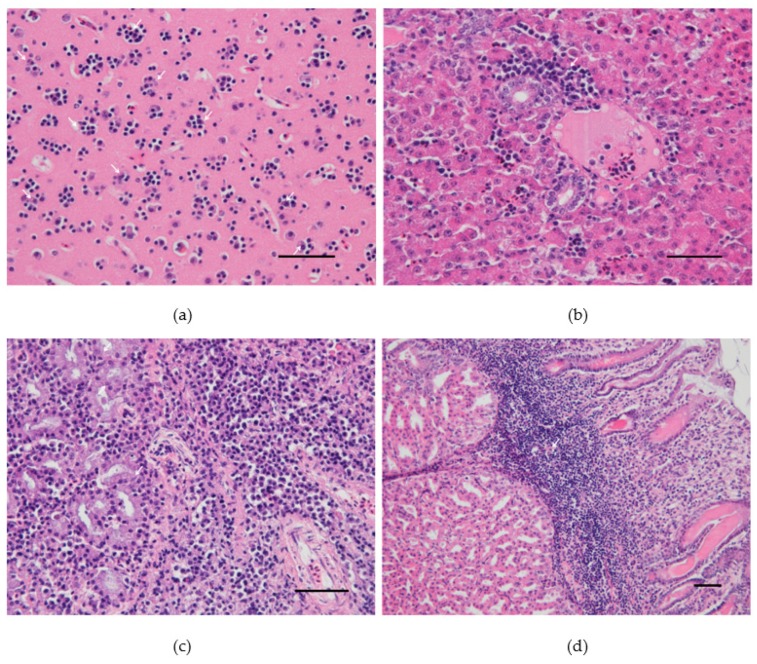
Pathological findings in the canary number 4 experimentally infected with 10^6^TCID_50_ of Usutu virus: (**a**) Cerebral cortex. Satellitosis: multiple foci of neuroglia around degenerating/apoptotic neurons. (**b**) Liver. Periportal hepatic inflammation: accumulation of lymphocytes, plasma cells, heterophils, and macrophages mostly around the portal area. (**c**) Lachrymal gland. Necrotic epithelial cells and massive lymphocytic and plasmacytic infiltrations within the interstitium. (**d**) Proventriculus. Marked lymphoplasmacytic and histiocytic infiltrates in the lamina propria. Hematoxylin and eosin, Scale bars: 50 µm.

At necropsy, dead canaries had splenomegaly and pallor of the liver. Histopathological investigations revealed severe satellitosis, neuronal necrosis, apoptosis, and neuronophagia in the brain of the canary 4 from group A (Figure 2a). The same lesions were milder in the other two dead canaries (Figure S1). Other common lesions consisted of slight perivascular infiltrates of lymphocytes and plasma cells in the lungs, moderate mononuclear inflammation and necrosis, consistently present in the liver (Figure 2b and Figure S2) and very mild in the heart, and histiocytosis with moderate lymphoid depletion in the spleen. Acinar cell necrosis and infiltration of lymphocytes and plasma cells in the interstitium were found in the lachrymal glands of the canary 4 from group A (Figure 2c). The same canary presented macroscopic hemorrhage in the proventriculus, in which severe inflammation and necrosis were also seen microscopically (Figure 2d). Canary 4 from group B also presented similar lymphoplasmacytic and histiocytic infiltrates in the lamina propria of the proventriculus (Figure S3).

### 3.3. Virus Detection by Immunohistochemistry

**Figure 3:**
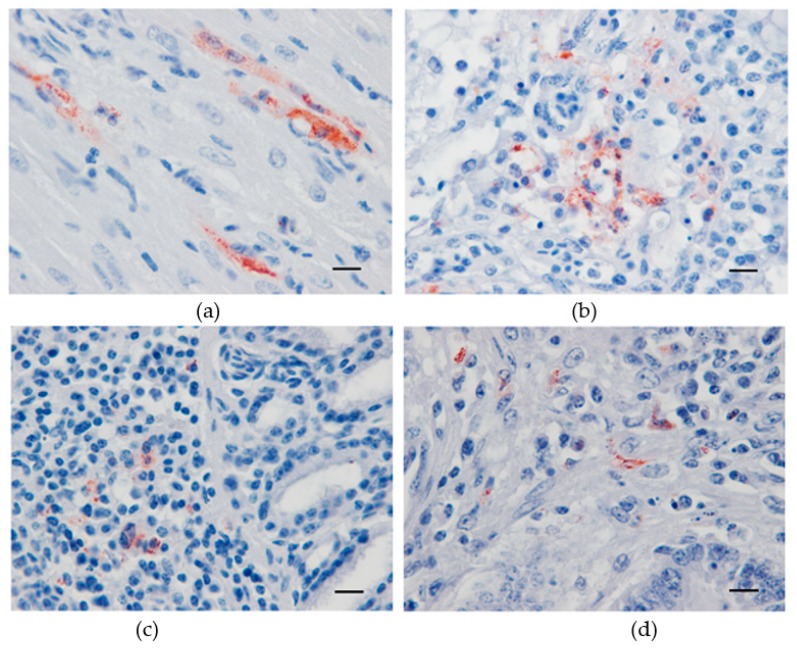
Immunohistochemical labeling of Usutu virus antigens in experimentally infected canaries using a mix of 4E9 and 4G2 anti-E protein monoclonal antibodies. Red-brown staining in antigen-positive cells from the heart (**a**), lung (**b**), lachrymal gland (**c**), and small intestine (**d**). Mayer hematoxylin counterstain, Scale bars: 10 µm.

All three lethally infected canaries exhibited USUV antigen immunolabeled cardiomyocytes (Figure 3a). In the liver of the canary which died at day 5 pi, numerous Kupffer cells were IHC-positive. Likewise, in the lung (Figure 3b), lachrymal gland (Figure 3c), and small intestine (Figure 3d), positive cells, presumably of a leukocytic origin, were randomly distributed. The brain, kidney, spleen, and skin were negative for USUV antigen.

### 3.4. Virus Detection by RT-qPCR

All birds, except controls, became infected with USUV, based on viral RNA detection by RT-qPCR in the serum as early as 1 dpi (Table 1). Very high RNAemia levels were found in the dead canaries during the course of their infection (Table 1). The USUV RNAemia showed a significant drop from 3.18–6.22 log10VRC/mL on day 1 pi to 0.7–2.8 log10VRC/mL on day 15 pi (*P* < 0.005) and did not statistically differ between the infected groups (*P* = 0.56). No detectable RNAemia was found in the control group on days 1, 3, 9, and 15 pi.

**Figure 4:**
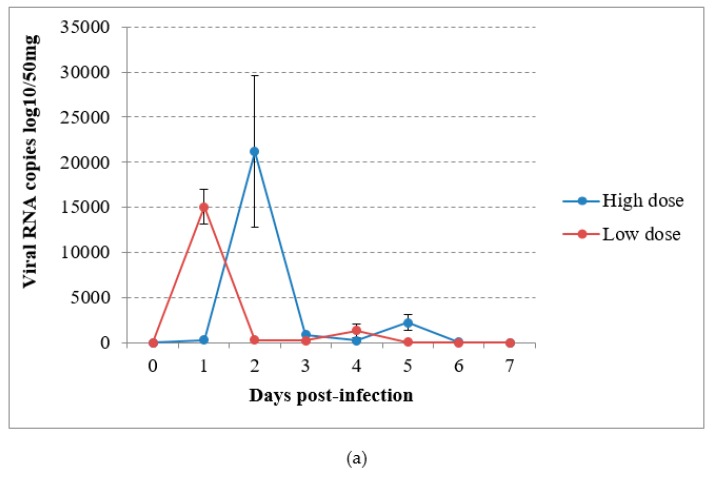

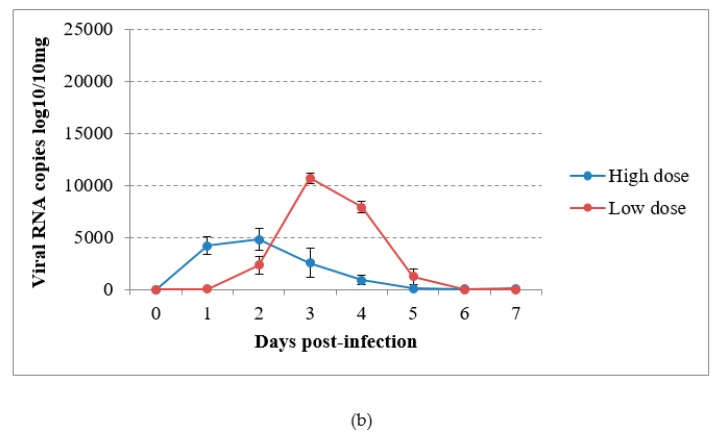
Viral RNA loads detected by RT-qPCR in (**a**) droppings and (**b**) immature feathers from canaries infected intraperitoneally with the Usutu virus.

In the virus-inoculated canaries, virus shedding was shown to occur from 2 to 5 dpi through the droppings in group A and from the first day to day 4 pi in group B, reaching a maximum at 1 or 2 dpi according to the group (Figure 4a). The detection of USUV RNA in immature feathers also lasted for 4 days, with a maximum of 4.02 log10 VRC/10mg recorded in group B (Figure 4b). No significant differences could be found in RNA shedding via the above-cited routes between groups (*P* = 0.53 and *P* = 0.614 respectively). The sham-inoculated group did not shed viral RNA via the feathers or droppings during the experiment.

All samples collected from the dead canaries at necropsy were USUV-positive by RT-qPCR, with high RNA amounts in their blood and tissues, as presented in Table 2.

### 3.5. Antibody Response to USUV

The absence of a previous USUV (and WNV) infection was ensured by negative ELISA results on blood samples collected before starting the experiment. Serum samples from all the surviving canaries 15 days post-infection, with one exception, showed a positive reaction in the ELISA (Table 3). Similarly, neutralizing antibodies to USUV were detected in all the surviving USUV-challenged canaries to 15 dpi, except the canary 2 from group B (Table 3). The highest titer of neutralizing antibodies was recorded in one canary from group A (1:80). On day 9 pi, the three birds with a sufficient amount of sera for SN testing (canary 1 from group A and canaries 1 and 4 from group B) all presented an antibody titer of 1:20. Serum samples were in insufficient amounts for antibody response assessment on day 3. The control group remained serologically negative for USUV infection until the end of the experiment.

## 4. Discussion

In the present study, we questioned the susceptibility of domestic canaries to USUV. To our knowledge, this is the first report of experimental infection with this virus in a passerine. After their injection with two different doses of USUV, three out of the ten infected birds succumbed to infection, in contrast to a high mortality rate (5/5) reported after 5 days of their challenge with as few as 10 PFU of WNV [28]. This suggests that USUV is less pathogenic for domestic canaries than WNV. The strain of USUV used in this infection could, however, be less virulent compared with the original strain (before cell passaging) or other genetically distinct strains. Thus, additional experimental infections should be conducted using different USUV strains to draw a general conclusion regarding WNV superior lethality in canaries. In natural conditions, infection with USUV could have a greater impact on this species, since needle infection may fail to recapitulate the full biological parameters of mosquito-borne transmission occurring in nature [36]. In fact, mosquito saliva released during an infectious blood meal was shown to increase the severity of infection for a variety of arthropod-borne flaviviruses [37,38,39]. Besides, the intradermal injection of the virus could have better mimicked the natural injection route of USUV by the feeding mosquito [40]. The amount of USUV inoculated by mosquitoes into a host is currently unknown. Depending on the mosquito species, the dose of WNV inoculated by one mosquito during a blood meal ranges between 10^3.4^ and 10^5.9^ PFUs [41]. Thus, the 10^3^ TCID_50_ and 10^6^ TCID_50_ challenge doses may have complied with the amount of USUV inoculated in the birds during a mosquito bite. However, in this experiment, morbidity and mortality rates did not statistically differ in a dose-dependent manner. In the three dead canaries, higher levels of RNAemia compared to the surviving ones were recorded during the infection, which might explain the fatal outcome in these birds, regardless of the infective dose. This result is similar to that described in the study of VanDalen et al. (2013), in which higher viremia was detected in American robins (*Turdus migratorius*) lethally infected with WNV, although inoculated with the lowest dose [42]. In addition, canaries inoculated with a higher dose of the virus did not develop significantly higher RNA load in their blood, similar to Reisen et al. (2006) [43] and VanDalen et al. (2013) [42]. For virus detection in the blood, we used the RT-qPCR technique, which is known to be more sensitive than titration assays [44]. We did not attempt virus titer measurement from the blood of the canaries; hence, their host competence (that is their aptitude to express sufficiently high viremia levels to infect naive mosquito vectors after a blood meal) is still unclear. Additional groups including individuals subjected to regular sample collection and weight measurement could have helped fill the gap in RNAemia and body mass evolution during the entire period of infection. Mortality rates were also preliminary and would need larger group sizes to be expressed in a relevant percentage.

Cloacal and/or oropharyngeal shedding of USUV was previously described following natural [45] or experimental [26] infection. Here, relatively high RNA shedding via the droppings (ranging from 2.3 to 4.3 log10 VRC/50mg) was found during 5 days following the infection. The infectiousness of the detected virus particles was not, however, assessed in cell culture. Nevertheless, the non-vector borne transmission of WNV was experimentally demonstrated via contaminated food, water, or air in birds [46,47,48,49,50] and similar alternative routes for USUV transmission deserve further investigations using this avian model. RNA shedding via droppings was unexpectedly detected one day earlier in the group inoculated with a low dose. This could be explained by the collection technique of droppings from cages, which implied random sampling of canaries rather than a systematic sampling of each bird. For this reason, the amount of RNA detected may have not indicated the mean RNA shedding in the infected group. Cloacal swabs could offer a more standardized method to study RNA shedding kinetics via droppings and permit its correlation with the RNAemia levels in future experiments. The presence and persistence of viral load in feather pulp were found in many bird species following flaviviral infection [44,51,52] and were suspected to contribute to direct transmission via feather picking [44]. Detection and amplification of the Israel turkey meningoencephalitis virus (a mosquito-borne flavivirus pathogenic for turkeys) from feathers was even proposed for evaluation of proper administration of live vaccines [53]. Our work is the first to demonstrate possible USUV RNA shedding via birds’ immature feathers in the early stages of infection. Whether feathers are able to disseminate infectious viruses in the environment or not and whether RNAemia levels are correlated to the amount of virus shed via feathers are still unsolved.

USUV pathogenesis in the lethally infected canaries entailed early onset of viremia, followed by a rapid viral invasion of all systems, as the virus was detected by RT-qPCR in all organs examined, including the brain, heart, liver, spleen, skin, and kidney. This systematic infection is similar to that reported in naturally infected birds [13]. Gross lesions in the present study included splenomegaly, pallor in the liver, and hemorrhage in the proventriculus, as described in spontaneous USUV infections [18]. Besides, similarly to those previously reported in naturally infected birds [18], severe inflammation and necrosis in multiple organs, including the brain were microscopically observed in the canary dead at day 5 pi. However, neurological manifestations and abundant USUV antigen were lacking. We also commonly found negative IHC staining in brains from naturally infected birds in Belgium [15]. Thus, death resulted more likely from multi-systemic failure than a pure neurologic disease, in a similar manner described for WNV infection in highly susceptible species [54,55]. Similarly, mild microscopic lesions and USUV antigen distribution patterns in the other two lethally infected canaries were inconsistent with the high RNA amounts in their organs and blood. Consequently, a part of the RNA detected by RT-qPCR could have been simply circulating in the blood. The mechanism leading to the death of these canaries remains unclear. However, the heart seems to be highly affected with viral replication, as virus antigens were systematically detected by IHC in the myocardium. 

The dissemination of USUV to the eye was here shown by RT-qPCR. This is consistent with the detection of abundant USUV antigens within the retina previously reported in experimentally infected goose embryos [25]. The necrotic and inflammatory changes in the lachrymal gland of one dead canary, along with diffuse lymphoplasmacytic infiltration, could have resulted in a lack of secretory activity and contributed to ocular disease. Visual impairment and ocular lesions were described following bird infection with other flaviviruses [56,57]. Vision assessment should be performed in future experimental infections with USUV. Further, the lachrymal gland of birds is part of the head-associated lymphatic tissue [58]. In the study by Chvala et al. (2004), USUV was detected in macrophages and dendritic cells of naturally infected blackbirds [18]. Together, these results demonstrate that USUV may target the immune cells in birds, which could play an important role in the spread of the virus in a wide variety of tissues, as described for WNV [59] and Tembusu virus [60] (a mosquito-borne flavivirus pathogenic for certain waterfowl birds). 

Two weeks after the experimental infection, almost all the surviving canaries showed a humoral response and specific neutralizing antibodies against USUV, which further demonstrates their susceptibility to USUV infection. Nevertheless, a single specimen from the group infected with the lower dose of USUV presented a doubtful serological response using the ELISA examination and an undetectable neutralizing antibodies titer by our technique. This finding could be due to a certain heterogeneity in the genetic background of the outbred canaries used in this experiment, which mimics the very specific host-pathogen interaction that has been described in nature ([13], Supplementary Materials Appendix 1). Experimental infections of several bird species with WNV have shown that the rise of antibodies against WNV occurs between five and 10 dpi [61]. Studies addressing the time-course of antibody response and sterilizing immunity against the USUV challenge should be conducted using this avian model. In addition, whether neutralizing antibodies to USUV could confer resistance to the infection by multiple flaviviruses from the JEV serocomplex, based on the cross-protection between these viruses [62,63,64] should be assessed. This information could be useful in the design of a broad-spectrum vaccine to protect birds against lethal infection (e.g., with WNV and USUV) and/or limit viral amplification (e.g., of JEV or St. Louis encephalitis virus) in these reservoir hosts.

## 5. Conclusions

In summary, we established that canaries are susceptible to USUV infection and can shed viral RNA via droppings and feathers. Further, we showed that USUV-associated mortality was not necessarily correlated to a pure neurological disease. These findings match those observed in European blackbirds and other *Passeriformes* when naturally infected with USUV. Further studies in canaries using other USUV strains circulating in Europe might contribute to a larger understanding of USUV pathogenesis. In addition, alternative transmission routes of USUV can be assessed using this avian model.

## Figures and Tables

**Table 1:** Usutu virus RNA (expressed in log10 viral RNA copies mL^−1^) detected by RT-qPCR in the serum of experimentally infected canaries.

			Days Post-Infection.				
	Canary	1	3	5	7	9	15
**Group A (10^6^TCID_50_)**	1	5.36 ± 0.01	5.21 ± 0.11			4.73 ± 0.1	0.37 ± 0.01
	2	7.01 ± 0.02	6.34 ± 0.03			7.01 ± 0.3	
	3	5.99 ± 0.06	5.23 ± 0.04			Insuff.	3.71 ± 0.24
	4	6.34 ± 0.03	6.73 ± 0.01	7.95 ± 0.08			
	5	5.50 ± 0.12	5.69 ± 0.01			3.83 ± 0.02	1.64 ± 0.21
**Group B (10^3^TCID_50_)**	1	7.13 ± 0.03	6.34 ± 0.03			2.81 ± 0.25	1.76 ± 0.23
	2	4.38 ± 0.01	5.80 ± 0.01			Insuff.	2.09 ± 0.29
	3	6.01 ± 0.02	5.83 ± 0.04			2.37 ± 0.4	1.6 ± 1.38
	4	7.13 ± 0.08	7.33 ± 0.01		8.20 ± 0.21		
	5	4.38 ± 0.03	5.96 ± 0.07			1.95 ± 0.52	2.00 ± 0.24

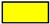
 Dead canary; Insuff. = Insufficient volume.

**Table 2:** USUV RNA loads in domestic canaries which succumbed to the experimental infection with USUV as determined by RT-qPCR and expressed in log10 viral RNA copies.

	Samples	Blood	Brain	Liver	Eye	Feathers	Lung	Kidney	Intestine
Bird									
**Group A**									
Canary 4		5.39 ± 0.31	7.15 ± 0.09	9.05 ± 0.19	7.18 ± 0.15	4.32 ± 0.09	9.12 ± 0.21	6.22 ± 0.14	6.62 ± 0.02
Canary 2		4.60 ± 0.81	6.19 ± 0.03	7.40 ± 0.50	3.73 ± 0.02	3.05 ± 0.62	7.26 ± 0.1	7.48 ± 0.03	3.86 ± 0.04
**Group B**									
Canary 4		7.30 ± 0.41	7.77 ± 0.01	5.05 ± 0.41	3.91 ± 0.3	3.32 ± 0.11	3.41 ± 0.07	4.53 ± 0.13	7.54 ± 0.10

**Table 3:** USUV-challenged canaries analyzed for antibodies against USUV on day 15 post-infection using the serum neutralization technique.

Surviving Birds		ELISA	Neutralizing Antibodies
**Group A (10^6^TCID_50_)**	1	+	**1:80**
	2	+	**1:20**
	3	+	**1:40**
**Group B (10^3^TCID_50_)**	1	+	**1:20**
	2	D	**<1:5**
	3	+	**1:40**
	4	+	**1:20**

D: Doubtful.

## Supplementary Materials

The following are available online at https://www.mdpi.com/1999-4915/12/2/164/s1, Figure S1: Cerebral cortex of canary 4 (a, low dose) and canary 2 (b, high dose). Satellitosis: Foci of neuroglia around degenerating/apoptotic neurons. Figure S2: Liver of canary 2 (high dose). (a) Periportal hepatitis (b) Focal hepatitis and necrosis. Figure S3: Proventriculus of canary 4 (low dose). Lymphoplasmacytic and histiocytic infiltrates in the lamina propria. Scale bars: 50 µm.
